# Idiosyncratic Mòjiāng virus attachment glycoprotein directs a host-cell entry pathway distinct from genetically related henipaviruses

**DOI:** 10.1038/ncomms16060

**Published:** 2017-07-12

**Authors:** Ilona Rissanen, Asim A. Ahmed, Kristopher Azarm, Shannon Beaty, Patrick Hong, Sham Nambulli, W. Paul Duprex, Benhur Lee, Thomas A. Bowden

**Affiliations:** 1 Division of Structural Biology, Wellcome Trust Centre for Human Genetics, University of Oxford, Roosevelt Drive, Oxford, Oxfordshire OX3 7BN, UK; 2 Division of Infectious Disease, Boston Children’s Hospital, Boston, Massachusetts 02115, USA; 3 Icahn School of Medicine at Mount Sinai, One Gustave L. Levy Place, #1124, New York, New York 10029, USA; 4 Boston University School of Medicine, Boston, Massachusetts 02118, USA

## Abstract

In 2012, cases of lethal pneumonia among Chinese miners prompted the isolation of a rat-borne henipavirus (HNV), Mòjiāng virus (MojV). Although MojV is genetically related to highly pathogenic bat-borne henipaviruses, the absence of a conserved ephrin receptor-binding motif in the MojV attachment glycoprotein (MojV-G) indicates a differing host-cell recognition mechanism. Here we find that MojV-G displays a six-bladed β-propeller fold bearing limited similarity to known paramyxoviral attachment glycoproteins, in particular at host receptor-binding surfaces. We confirm the inability of MojV-G to interact with known paramyxoviral receptors *in vitro*, indicating an independence from well-characterized ephrinB2/B3, sialic acid and CD150-mediated entry pathways. Furthermore, we find that MojV-G is antigenically distinct, indicating that MojV would less likely be detected in existing large-scale serological screening studies focused on well-established HNVs. Altogether, these data indicate a unique host-cell entry pathway for this emerging and potentially pathogenic HNV.

## Abstract (web summary)

The attachment glycoprotein (HNV-G) of henipaviruses interacts with host receptors at the cell surface and is a major determinant of species tropism. Here the authors provide structural and functional evidence that the emergent henipavirus, Mòjiang virus, uses an entry mechanism that is independent of known paramyoxviral cellular receptors.

Three miners succumbed to severe pneumonia in Mòjiāng Hani Autonomous County, China, in June 2012. Investigation of bat, rat and musk shrew samples from within the cave, where the victims had most probably contracted the disease, led to the discovery of a novel rat-borne paramyxovirus, Mòjiāng virus (MojV)^1^. Analysis of the MojV genome reveals that the paramyxovirus is closely related to viruses within the genus *Henipavirus*, an emergent zoonotic group of bat-borne pathogens distributed throughout Asia, Australia, Central America and Africa^2^,^3^,^4^. Henipaviruses (HNVs) comprise some of the most virulent known paramyxoviruses; therefore, the identification of a new species closely related to members of this genus is of significant cause for concern.

Asiatic Nipah virus (NiV) and Hendra virus (HeV) are prototypic members of the HNV genus and upon zoonotic spillover into human populations cause acute encephalitis and respiratory illness, with some reported case-fatality rates exceeding 90% (refs 2, 3). Viruses belonging to additional >20 putative HNV clades have been detected in bat reservoirs in Africa and the near-complete genome of a Ghanaian bat HNV isolate has been sequenced. This isolate, previously termed as Ghana virus or Gh-M74a virus^4^,^5^, is now known as Kumasi virus (KV)^6^. It is unknown whether African HNV infection results in human disease^7^. However, there is serological evidence of HNV spillover events into humans in Africa and it has been proposed that these viruses may contribute to the prevalence of undiagnosed or misdiagnosed encephalitis^4^,^7^,^8^.

Paramyxovirus entry into a host cell requires two concerted steps: (i) viral attachment to a proteinaceous host cell surface receptor or sialic acid moiety and (ii) receptor-mediated fusion of the viral and host cell membranes^9^. Viral attachment is a primary determinant of host cell tropism and is initiated by a haemagglutinin-neuraminidase (HN) glycoprotein, haemagglutinin (H) glycoprotein or attachment (G) glycoprotein, depending on the virus. Avula-, respiro-, rubula-, ferla-10 and aquarespiroviruses^11^ express HN glycoproteins which recognize sialic acids on glycoproteins and glycolipids. H and G proteins, on the other hand, are expressed by morbilliviruses and HNVs, respectively, and recognize proteinaceous cell surface receptors. The H glycoprotein from the prototypic morbillivirus, measles virus (MV), targets CD150 (also known as signalling lymphocytic activation molecule F_1_) and nectin-4 cell surface receptors^12^,^13^. G glycoproteins from HNVs recognize at least one of the ubiquitously expressed ephrinB2 and ephrinB3 receptors^3^,^14^,^15^,^16^,^17^.

Despite the diverse host receptors targeted, paramyxoviral HN, H and G proteins exhibit a conserved molecular architecture comprising an amino-terminal cytoplasmic tail, a single transmembrane helix, a stalk region and a carboxy-terminal six-bladed β-propeller^18^,^19^,^20^,^21^,^22^,^23^,^24^. The paramyxoviral six-bladed β-propeller is integral in establishing host tropism and is structurally adapted to the natural host receptor^25^. For paramyxoviral HN glycoproteins, the sialic acid-binding site is located at a deep, central pocket at the top face of the six-bladed β-propeller^26^. The morbillivirus H glycoprotein binding sites for CD150 and nectin-4, on the other hand, overlap extensively at the side of the β-propeller^27^,^28^. Lastly, the ephrin binding site of HNV-G is located at the top face of the β-propeller and this region exhibits an elevated level of sequence conservation across the genus^14^,^17^,^29^,^30^, presumably due to a functional constraint to maintain ephrin binding.

The capability of the paramyxoviral β-propeller to accommodate varied proteinaceous and glycan receptors provides a structural rationale for differential cellular and species tropism^16^,^25^. As such, an understanding of how this scaffold is utilized for receptor recognition by newly emergent and potentially dangerous paramyxoviruses is of significant interest and offers a framework for understanding determinants of host-cell infection and zoonosis. Here, through analysis of MojV-G, we establish the pathobiological independence of MojV from other paramyxoviruses. Although MojV-G displays a six-bladed β-propeller fold characteristic to paramyxoviral attachment glycoproteins, our analyses also show that MojV-G is antigenically, structurally and functionally distinct from all other characterized paramyxoviruses. We attribute these differential properties to the absence of a requirement for MojV to target established paramyxoviral receptors. These data indicate an idiosyncratic host cell entry mechanism for this emergent paramyxovirus.

## Results

### MojV-F and -G mediate fusion in rodent and human cells

**Figure 1:**
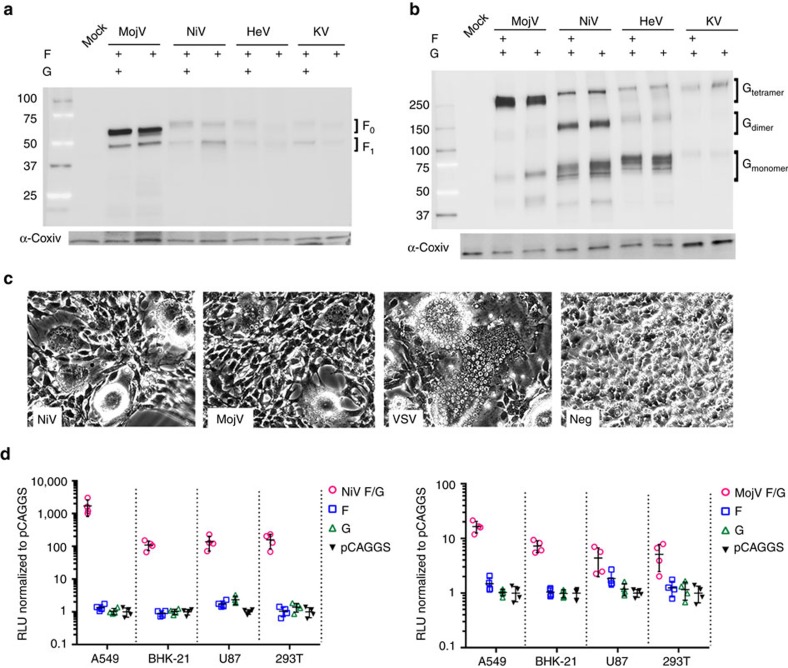
MojV-F and -G glycoproteins are functional and mediate cellular fusion on both rodent and human cell types. (**a**) Expression of MojV-F, NiV-F, HeV-F and KV-F in BSR-T7 cells in the presence or absence of the cognate G glycoprotein. F proteins were expressed with a C-terminal AU1 tag and cell lysates were subjected to Western blot analysis under reducing conditions with a rabbit anti-AU1 antibody. The F_0_ precursor and proteolytically cleaved F_1_ are annotated. (**b**) Expression of MojV-G, NiV-G, HeV-G and KV-G in BSR-T7 cells in the presence or absence of the cognate F protein. G proteins were expressed with a C-terminal HA epitope tag and cell lysates were subjected to western blot analysis under non-reducing conditions with a rabbit anti-HA antibody. Putative monomeric, dimeric, and tetrameric species are annotated. (**c**) Co-expression of MojV-F and MojV-G forms visible syncytia in BSR-T7 cells. Syncytia formed by expression of vesicular stomatitis virus-G and co-expression of NiV-F and -G are positive controls. (**d**) A quantitative heterologous fusion assay shows that MojV-F and -G-mediated fusion occurs with a variety of target cells of diverse species and tissue origins. HNV-F/G expression constructs were transfected into BSR-T7 cells and overlaid with a variety of target cells (A549, BHK-21, U87 and 293T cells) encoding T7-driven *Gaussia* luciferase. *Gaussia* luciferase activity, read in RLU (relative light units), is a marker for HNV-F/G-mediated cell-cell fusion. Maximal levels of fusion activity for each cell line are shown for NiV-F/G (left panel) and MojV-F/G (right panel). NiV-F/G-mediated fusion are included as positive controls, while cells transfected with MojV- and NiV-F and -G alone or empty vector (pCAGGS) are included as negative controls to set background levels of *Gaussia* luciferase activity. Fold change in RLU, as compared with empty vector fusion, is shown for each F/G combination and the F and G alone controls. Data shown are the individual data points from four independent biological replicate experiments (*n*=4). Horizontal dashes represent the mean from the four replicates and the error bars represent s.d.

We sought to determine whether published MojV-F and -G sequences give rise to functional proteins. Full-length MojV-F and MojV-G open reading frames were cloned into mammalian expression vectors with an AU1 or HA epitope tag at the C terminus, respectively, as previously described^15^,^30^. Immunoblotting revealed that MojV-G and MojV-F display expression profiles analogous to cognate NiV, HeV and KV glycoproteins: MojV-F undergoes proteolytic cleavage from an *F*_0_ glycoprotein precursor to a disulfide linked, mature *F*_1_/*F*_2_ complex (Fig. 1a) and MojV-G forms higher-order oligomers, constituting putative dimeric and tetrameric species, under non-reducing conditions (Fig. 1b).

**Figure 2:**
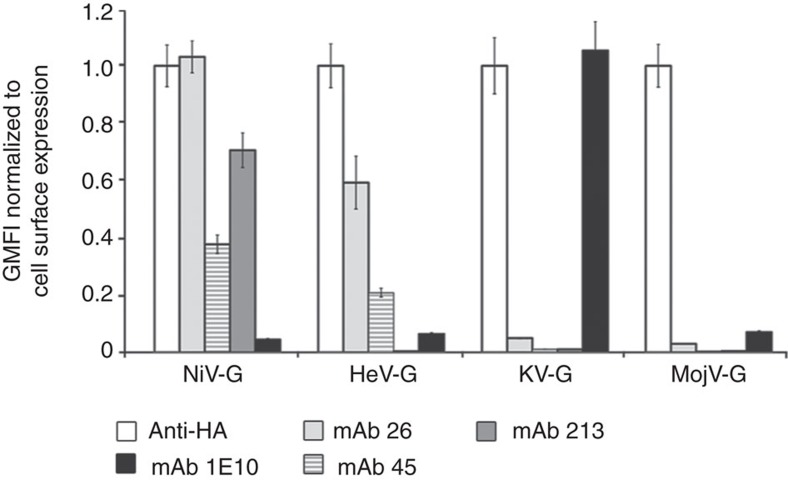
MojV-G is antigenically distinctive from Asiatic and African HNVs. Cross-reactivity of Asiatic HNV (NiV/HeV)-specific monoclonal antibodies (monoclonal antibodies 26, 45 and 213) and African HNV (KV)-specific antibody mAb 1E10 with MojV-G. HEK 293T cells were transfected with expression vectors encoding HA-tagged MojV-G, NiV-G, HeV-G and KV-G. Monoclonal antibody binding was normalized to HNV-G cell-surface expression as detected by anti-HA staining. Neither the Asiatic HNV-reactive nor the African HNV-reactive monoclonal antibodies exhibited cross-reactivity with MojV-G. Data are shown as the mean normalized geometric mean fluorescence intensity (GMFI) of three independent biological replicate experiments (*n*=3, error bars represent s.e.m.). Each individual GMFI value was generated from a composite of 10,000 collected events.

**Figure 3:**
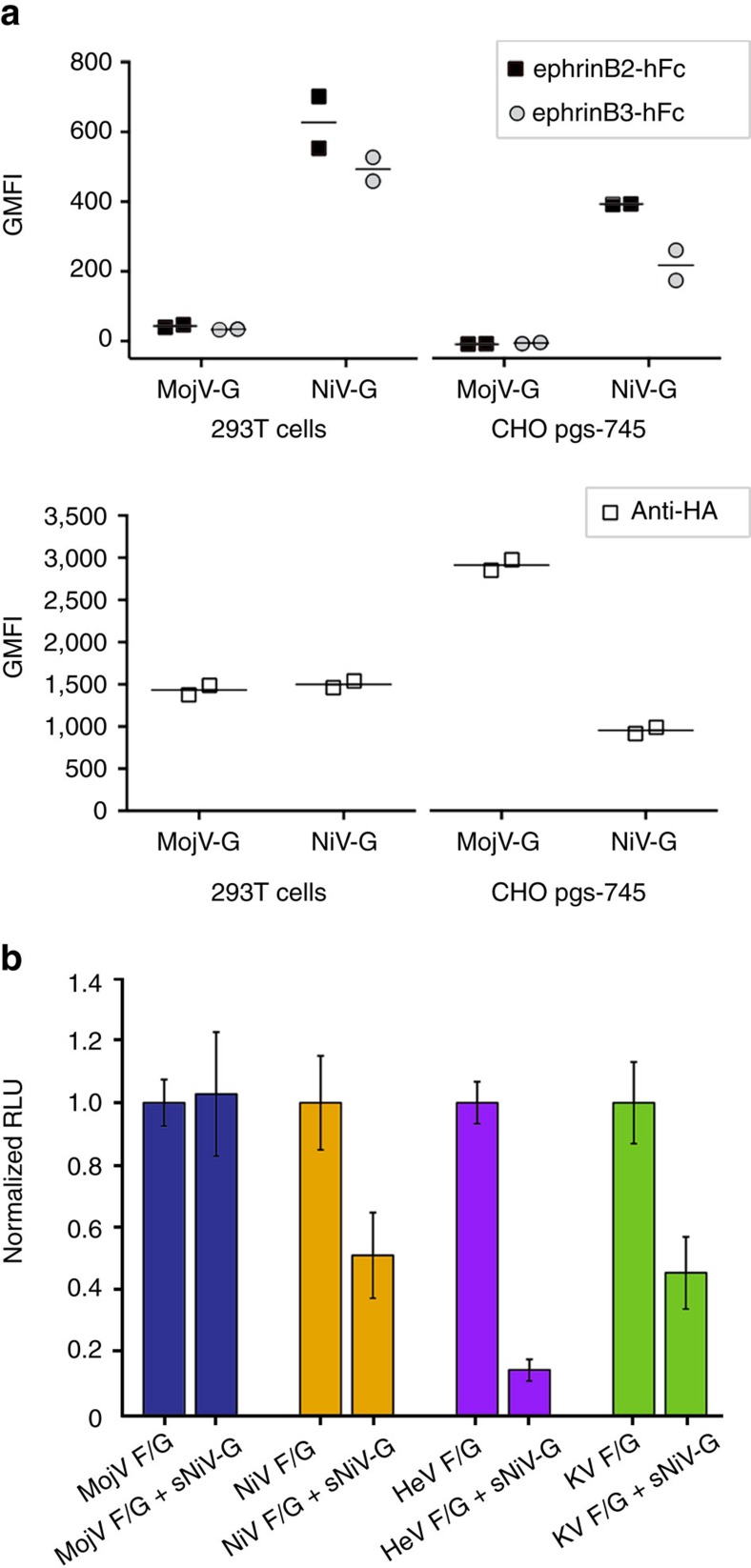
MojV-G does not bind to human ephrinB2 or ephrinB3. (**a**) Binding of soluble Fc-tagged, human ephrinB2 and ephrinB3 to MojV-G and NiV-G expressed with a C-terminal HA epitope tag in HEK 293T or CHO pgsA-745 cells. Background levels of soluble ephrinB2/B3 binding and anti-HA binding were measured from cells transfected with empty vector (pCAGGS). As expected, both ephrinB2 and B3 demonstrate high-affinity to NiV-G. MojV-G is present at the cellular surface in similar or higher levels than NiV-G; however, neither ephrinB2 nor ephrinB3 interacted with MojV-G. Data shown are the background-reduced, individual geometric mean fluorescence intensity (GMFI) values from two independent biological replicate experiments (*n*=2). Horizontal lines represent the mean of the two replicate values. Each individual GMFI value was generated from a composite of 10,000 collected events. (**b**) Soluble NiV-G does not inhibit MojV-F/G-mediated fusion. Soluble NiV-G-hFc was pre-incubated with BHK target cells and efficiently inhibited fusion with BSR-T7 effector exhibiting NiV-F/G, HeV-F/G and KV-F/G, but not MojV-F/G. *Gaussia* luciferase activity, RLU read in triplicate, was used as a marker for cell-to-cell fusion. Measurements are shown normalized according to the signal obtained when cells were incubated with hFc instead of NiV-G-hFc, establishing standard level of fusion for each experiment. Data shown are mean values from five independent biological replicate experiments (*n*=5, error bars represent s.d.).

We observed syncytia formation after co-expression of MojV-F and MojV-G, a strategy previously established for NiV-F/G and HeV-F/G^31^. Although syncytia did not form when MojV-G and MojV-F were expressed individually, co-expression of these glycoproteins led to syncytia in rodent-derived BSR-T7 cells (Fig. 1c). Indeed, expression of MojV-F/G in BSR-T7 cells in a quantitative heterologous cell-to-cell fusion assay revealed that A549 (human alveolar basal epithelial), BHK (baby hamster kidney), U87 (human glioblastoma) and HEK (human embryonic kidney) 293T cells support MojV-F/G glycoprotein-mediated fusion, albeit at lower levels than that observed for NiV-F/G glycoproteins (Fig. 1d). The wide range of cells observed to be permissive to MojV-F/G-mediated fusion is consistent with the proposed zoonotic potential of MojV^1^. Interestingly, MojV-F/G-mediated fusion is significantly less robust than that mediated by cognate NiV glycoprotein counterparts (Fig. 1d), even though MojV-F appears to be similarly cleaved and MojV-G is expressed well on the cell surface (see Figs 2 and 3 below).

### Antigenically distinct MojV-G does not use ephrinB2/B3

Sequence-based phylogenies place MojV within the *Henipavirus* genus^1^,^6^,^25^. However, MojV-G is divergent, exhibiting only 20–24% sequence identity with known HNV attachment glycoproteins. We sought to determine whether MojV-G, is antigenically related to Asiatic (NiV/HeV) or African clades (KV) of HNVs and found that previously reported antibodies against NiV/HeV-G, and an antibody derived in this study against KV-G (termed 1E10), failed to cross-react with MojV-G, despite clear expression of MojV-G on the cell surface (Fig. 2). The antigenic distinctiveness of MojV-G is in-line with the divergent relationship of MojV with respect to other HNVs.

We then sought to investigate whether MojV-G could interact with ephrinB2 and ephrinB3 *in vitro*, as this has been observed to be a salient feature of both African and Asiatic HNV-Gs^32^. Although soluble ephrinB2 and ephrinB3 clearly bound to HEK 293T and Chinese hamster ovary (CHO) pgsA-745 cells transfected with HA-tagged NiV-G, no significant binding was observed to cells similarly transfected with HA-tagged MojV-G (Fig. 3a) even though MojV-G was expressed on the cell surface at least as well as NiV-G, and in the case of CHO-pgsA-745 cells, even better than NiV-G. Furthermore, although soluble NiV-G-hFc was able to inhibit NiV, HeV and KV F/G-mediated fusion to variable extents in a heterologous fusion assay, it was not able to inhibit MojV-F/G-mediated fusion (Fig. 3b). Lastly, in the same fusion assay format, CHO pgsA-745 cells expressing ephrinB2 demonstrated robust fusion with BSR-T7 effector cells expressing NiV-F/G or KV-F/G, but not BSR-T7 effector cells expressing MojV-F/G (Supplementary Fig. 1). Altogether, these data indicate that MojV-G is functionally distinct from canonical HNVs and does not utilize prototypic ephrinB2/B3-dependent attachment and entry pathways.

### Structure of MojV-G

**Figure 4:**
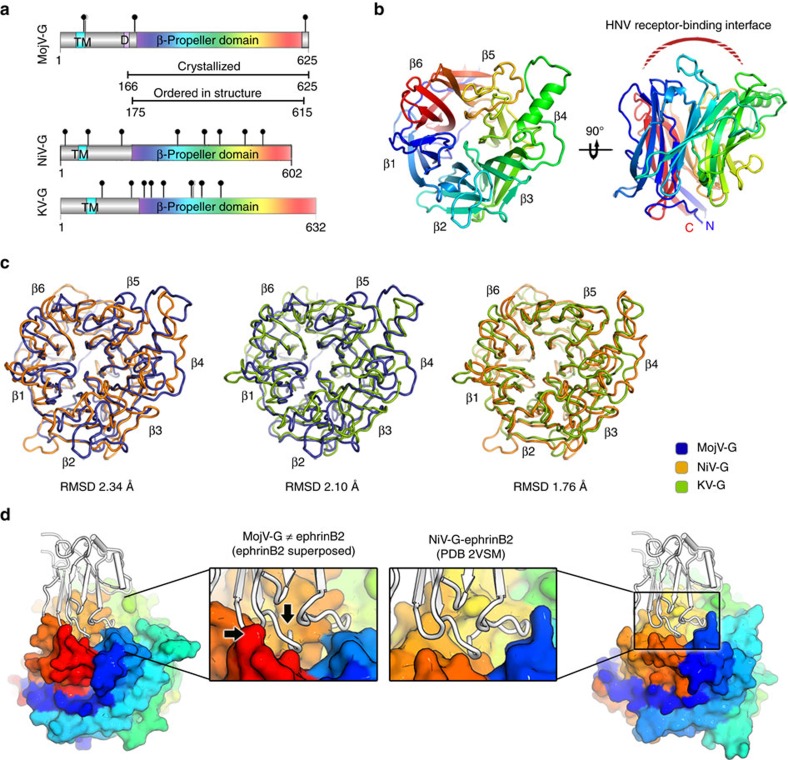
Structural analysis of MojV-G. (**a**) Domain schematics of MojV-G, NiV-G and KV-G indicate the locations of the transmembrane domain (TM), aspartic acid-rich domain D in MojV-G, β-propeller domain and *N*-linked glycosylation sequons (black spikes). (**b**) Crystal structure of the MojV-G β-propeller domain (cartoon representation, coloured rainbow with the N terminus in blue and the C terminus in red). (**c**) Structural comparison of MojV-G with other HNV-G glycoproteins. Overlays of MojV-G (coloured blue) with the equivalent domains from NiV-G (orange, PDB 2VWD) and KV-G (green, PDB 4UF7), rendered in Cα trace representation. (**d**) Comparison of MojV-G with the crystal structure of the NiV-G β-propeller domain in complex with ephrinB2 (PDB 2VSM), right, reveals that MojV-G lacks the ephrin binding pocket found in classical HNVs. Indeed, the interaction between MojV-G and ephrinB2/B3 would be obstructed (clashes indicated by black arrows) by the ‘closed’ arrangement of loops in first (blue) and fifth (orange) blades of the MojV-G β-propeller. NiV-G and MojV-G are rendered in a surface representation and coloured rainbow with the N terminus in blue and the C terminus in red. EphrinB2 is shown as a white ribbon.

We sought to determine whether the functional independence of MojV-G from established HNVs is reflected in the atomic structure. A construct encoding some residues from the putative MojV-G stalk region (residues 166–193), the β-propeller domain (residues 194–605) and the C-terminal extension (residues 606–625), was expressed in HEK 293T cells in the presence of an α-mannosidase inhibitor, kifunensine (Fig. 4a)^33^,^34^. Consistent with the exclusion of the putative cysteine-rich oligomerisation region of the stalk domain, size exclusion analysis revealed that MojV-G was monomeric in solution. Following deglycosylation with endoglycosidase F_1_ (EndoF_1_), MojV-G was crystallized and the structure was determined to 1.94 Å resolution (Fig. 4b and Table 1).

MojV-G exhibits a six-bladed β-propeller fold, characteristic of *Paramyxovirinae* attachment glycoproteins, with each blade of the propeller composed of four antiparallel β-strands (Fig. 4b). The β-propeller fold is stabilized by seven disulfide bonds, all of which are conserved among HNVs (Supplementary Fig. 2). Two nearly identical molecules of MojV-G were observed in the asymmetric unit (0.33 Å root-mean-square (RMS) deviation over 412 aligned Cα atoms), although crystallographic contacts were not consistent with the formation of higher-order MojV-G oligomers, such as those observed in HeV-G^18^ or other paramyxoviruses^19^,^20^,^21^,^22^,^23^,^35^. The majority of residues corresponding to the C-terminal extension of MojV-G were disordered in the crystal (residues 616–625). However, a portion of the crystallized N-terminal stalk region was clearly visible (residues 175–193) and folds underneath the sixth blade of the β-propeller domain in a conformation analogous to that observed in full-length Newcastle disease virus (NDV) HN glycoprotein and parainfluenza 5 HN (PIV5-HN) glycoprotein ectodomain structures (Supplementary Fig. 3)^19^,^20^. The similar positioning of this region among these structures is consistent with the conserved tertiary organization of the paramyxoviral glycoprotein spike.

Unlike other paramyxovirus attachment glycoproteins, which are glycosylated throughout the β-propeller^18^,^19^,^20^,^21^,^22^,^23^,^24^,^35^,^36^,^37^,^38^,^39^,^40^,^41^,^42^,^43^,^44^,^45^,^46^, the ectodomain of MojV-G encodes only four *N*-linked glycosylation sequons, all of which are located outside the β-propeller domain (Fig. 4a). Two of these sites, Asn189 and Asn619, were included in the crystallized MojV-G construct (residues 166–625). Enzymatic deglycosylation of this construct with EndoF_1_ induced a mobility shift in SDS-PAGE, indicating that at least one of these sites bears an *N*-linked glycan (Supplementary Fig. 4a). As Asn619 is located at the disordered C terminus of our MojV-G structure, it is unknown whether this sequon is glycosylated. Asn189, on the other hand, is partially ordered in one of the two MojV-G molecules in the asymmetric unit (chain B). However, no electron density corresponding to an *N*-acetylglucosamine moiety extension was detected at this site (Supplementary Fig. 4b), indicating that either Asn189 is not glycosylated at a high occupancy level or that the associated glycan adopts multiple conformations in the crystal.

Regardless of whether Asn189 and/or Asn619 are glycosylated, it is highly unusual for a paramyxoviral attachment glycoprotein to lack *N*-linked glycans throughout the β-propeller domain proper, given their crucial role in virulence, host immune evasion, glycoprotein folding and assembly, and the activation of fusion cascades^36^,^37^,^38^,^47^,^48^,^49^. Therefore, the absence of *N*-linked glycans in the β-propeller domain appears to be a distinguishing feature of MojV-G, differentiating it not only from classical HNV-Gs^18^,^24^,^36^,^37^,^38^,^39^ but also HN^19^,^20^,^21^,^22^,^23^ and H^35^,^46^ glycoproteins and the glycoproteins of the recently emerged feline morbillivirus^40^,^41^,^42^, Mossman^43^, Nariva^44^ and Salem^45^ viruses. Our functional and structural characterization of the uniquely aglycosylated MojV-G β-propeller domain is suggestive that, in contrast to other paramyxoviral receptor-binding proteins, *N*-linked carbohydrates are unlikely to be required for native functionality or evasion of the host immune response.

### MojV-G is dissimilar to Paramyxoviral attachment proteins

The MojV-G six-bladed β-propeller fold is structurally dissimilar to all known attachment glycoproteins from members of the *Paramyxoviridae* family. Indeed, overlay analysis reveals that MojV-G exhibits only a slightly greater resemblance to HNV-G glycoproteins (2.1–2.3 Å RMS deviation upon superposition of equivalent Cα atoms, Fig. 4c) than sialic acid-binding HN glycoproteins found in members of the *Respirovirus* genus (RMS deviation of 2.3 Å from human parainfluenza virus type-3, (hPIV3), HN glycoprotein over 375 equivalent Cα atoms; PDB accession code 1V2I), *Rubulavirus* genus (RMS deviation of 2.5 Å from PIV5-HN over 366 equivalent Cα atoms; 1Z4Y) and *Avulavirus* genus (RMS deviation of 2.5 Å from NDV-HN glycoprotein over 367 equivalent Cα atoms; 1E8T). Notably, MojV-G also poorly overlays with morbillivirus H glycoproteins (RMS deviation of 3.1 Å from measles virus haemagglutinin (MV-H), over 282 equivalent Cα atoms; 2RKC). Indeed, we observe that paramyxoviral receptor-interacting surfaces exhibit some of the greatest levels of structural deviation with respect to MojV-G. For example, the top face of the MojV-G β-propeller lacks the distinctive ephrin binding pocket observed in NiV-G (Fig. 4d) and other HNVs, and exhibits very low sequence conservation with other HNVs at this region^25^. It also appears that the interaction between MojV-G and the primary ephrinB2/B3-binding interface would be sterically hindered by the ‘closed’ arrangement of loops in first and fifth blades of the MojV-G β-propeller (Fig. 4d). This provides a structural rationale for the lack of ephrinB2 usage (Fig. 3), suggesting that MojV is functionally distinct from HNVs in terms of receptor tropism.

**Figure 5:**
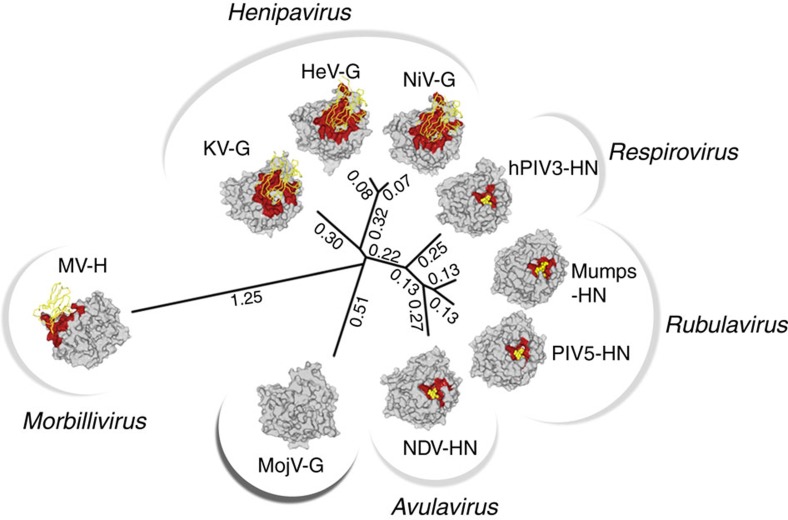
MojV-G is structurally independent from other paramyxovirus host-recognition proteins. A structure-based phylogeny of paramyxoviral receptor binding proteins, produced using SHP software^61^,^62^. Receptor-binding proteins are shown besides the tree and are rendered as surface representation (grey). All receptor-binding proteins, with the exception of the MojV-G, are shown in complex with their respective receptor molecules (yellow), with the receptor-binding interface coloured red. PDB accession codes for the structures shown are 2VSM (NiV-G), 2VSK (HeV-G), 4UF7 (KV-G), 1V3C (human PIV3-HN), 1Z4V (PIV5-HN), 5B2D (mumps-HN), 1E8V (NDV-HN) and 3ALW (MV-H).

These architectural differences are similarly evident upon structure-based phylogenetic analysis, which places MojV-G almost equidistant from all structurally studied genera (Fig. 5). In contrast to established paramyxoviral host-recognition glycoproteins, which cluster according to respective genera and cellular receptor usage^30^, the distal placement of MojV-G from HNV-G, H and HN glycoproteins indicates the absence of a functional and evolutionary constraint for MojV-G to utilize known paramyxoviral receptors.

**Figure 6:**
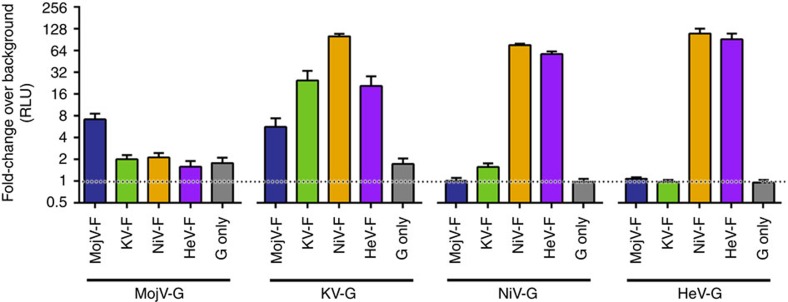
Homo- and heterotypic complementation of HNV-F and -G-mediated fusion as observed in a BSR-T7 effector/BHK target quantitative heterologous fusion assay. Fusion occurs when each paramyxovirus F glycoprotein is paired with its cognate G partner, albeit with different efficiencies. Fusion is most robust with NiV and HeV glycoproteins (rendered in orange and purple, respectively). KV-G (green) hetero-complements efficiently with HeV-F and NiV-F in addition to cognate KV-F. MojV-G did not complement any of the HNV-F proteins tested, corroborating the functional distance of MojV-G from the known HNV-G proteins. Hetero-complementation is observed between MojV-F and KV-G, indicating that some features of the more conserved MojV-F remain congruent with fusion triggering mechanism of HNVs. *Gaussia* luciferase activity, RLU read in triplicate, was used as a marker for cell-to-cell fusion. Background fusion level was measured from effector cells transfected with plasmid pCAGGS only and fold-change in RLU as compared with background fusion is shown for each F/G combination and G alone (negative control). Data shown are mean values from five independent biological replicate experiments (*n*=5, error bars represent s.e.m.).

We further illustrated the differential functionality of MojV and HNV glycoproteins by assessing whether HNV-F and HNV-G could heterotypically complement MojV-G and MojV-F, respectively. Although heterotypic complementation appears to occur even among distantly related HNVs^5^, MojV glycoproteins exhibited very limited levels of heterocomplementation, where no HNV-Fs were triggered by MojV-G and only KV-G was observed to heterocomplement MojPV-F (Fig. 6) to a minimal extent. Combined, these structural and functional data indicate that MojV utilizes differing receptor attachment and viral fusion pathways from classical HNVs.

### MojV-G does not bind human CD150

**Figure 7:**
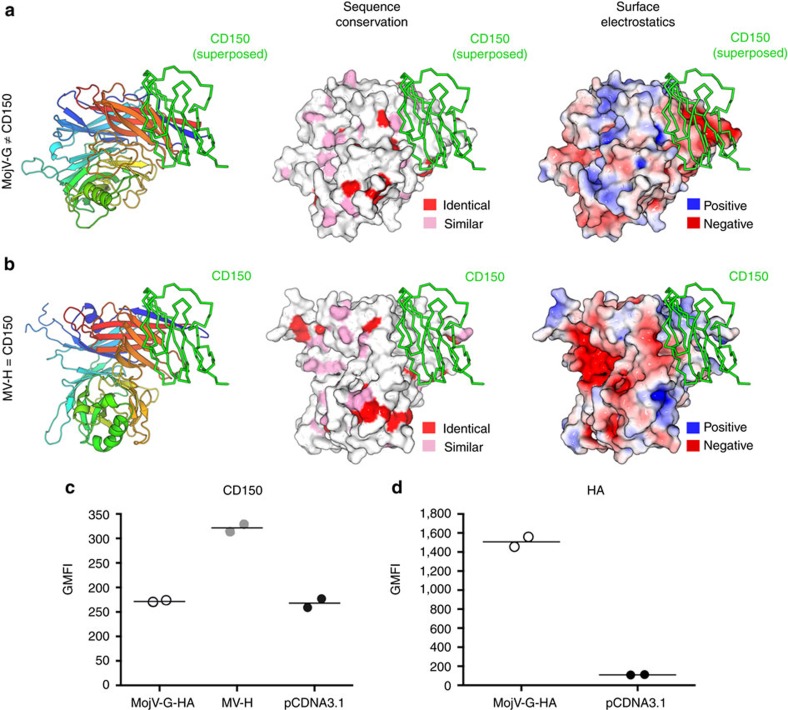
Host cell attachment of MojV-G is independent of the hCD150 receptor. (**a**) Structure of MojV-G shown as a cartoon and coloured as a rainbow, as in Fig. 4b. The relative position of the hCD150 binding site on the MojV-G β-propeller was determined by overlay of the MojV-G structure with the MV-H-hCD150 complex structure (PDB accession code 3ALZ), with hCD150 shown as a green ribbon. (middle) MojV-G is shown in surface representation and coloured according to sequence conservation with MV-H (UniProt accession number P08362). Residues coloured red are conserved, residues coloured pink are similar and residues coloured white are not conserved. (Right) Electrostatic surface potential of MojV-G, as calculated with the APBS^70^ extension within PyMOL (http://www.pymol.org/). MojV-G is shown in surface representation with electrostatic charges ramped from red (−5 kT e^−1^) to blue (+5 kT e^−1^). (**b**) Structure of the MV-H-hCD150 complex (PDB accession code 3ALZ). Sequence conservation and surface electrostatic potentials are shown as in **a**. (**c**) HEK 293T cells transfected with either MojV-G-HA, MV-H or empty vector control (pCDNA3). Cells were stained with hCD150-FLAG, counterstained with murine anti-FLAG (SIGMA) and goat anti-mouse-Alexa 647. Binding of hCD150 to cells was assessed by flow cytometry. (**d**) Confirmation of MojV-HA surface expression by anti-HA staining with cells transfected with pCDNA3 used as a control. Data shown in panels c and d are the individual geometric mean fluorescence intensity (GMFI) values from two independent biological replicate experiments (*n*=2). Horizontal lines represent the means of the two replicate values. Each individual GMFI value was generated from a composite of 10,000 collected events.

As MojV-G does not exhibit ephrinB2/B3 specificity, we investigated whether MojV-G could recognize other known H and HN glycoprotein receptors. Structural comparison of MojV-G with MV-H indicates that MojV-G is unlikely to recognize morbillivirus-specific cell surface receptors, such as CD150 (ref. 28) and nectin-4 (ref. 27). Upon overlay of MojV-G and MV-H, we note the absence of sequence conservation and significant charge differences at the CD150 receptor-binding site (Fig. 7a,b). There is also very limited sequence conservation with MojV-G at this region, where MV-H receptor-interacting residues are less than 20% conserved with MojV-G. To confirm that MojV-G does not utilize CD150, we performed a flow cytometry-based binding assay using soluble human CD150 (hCD150) to detect MojV-G or MV-H glycoprotein expressed in HEK 293T cells. Although soluble hCD150 avidly bound MV-H glycoprotein expressing cells, there was no detectable interaction between hCD150 and MojV-G (Fig. 7c,d). Differential characteristics of MojV-G and MV-H were further corroborated by immunostaining, where antibodies against MV-H failed to recognize MojV-G (Supplementary Fig. 5). Although we cannot preclude the possibility of MojV-G interacting with non-human CD150, especially given the relatively low level of sequence conservation in different organisms^25^, our data do not support a CD150-mediated entry mechanism into human cells.

### MojV-G lacks key sialic-acid binding residues

**Figure 8:**
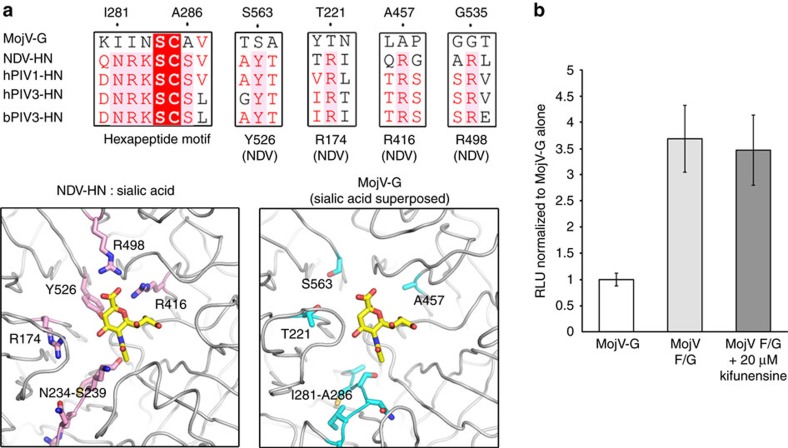
Sialic acid independence of MojV-G-mediated host cell attachment. (**a**) Above: Sequence alignment of MojV-G and HN proteins from NDV, hPIV-1, hPIV-3 and bovine PIV3 (bPIV3) reveals that MojV-G lacks the conserved motifs required for sialic acid binding and neuraminidase functionality. These sequence motifs include: (i) the hexapeptide NRKSCS, (ii) a tyrosine involved in neuraminidase activity and fusion promotion (Y526 in NDV), and (iii) a triarginyl cluster (R174, R416 and R498 in NDV)^26^. Below: structural comparison of the sialic-acid binding pocket of NDV-HN (left, PDB 1E8U) with MojV-G. The sialic acid ligand observed in the NDV-HN:sialic acid co-structure is rendered as yellow sticks (left) and is shown superposed in the equivalent position in MojV-G (right). Residues highlighted in the above alignment are shown as pink and cyan sticks in NDV-HN and MojV-G, respectively. (**b**) Kifunensine treatment of BHK target cells as high as 20 μM does not inhibit MojV-F/G-mediated fusion, indicating that MojV entry is sialic acid independent. *Gaussia* luciferase activity, RLU read in triplicate, was used as a marker for cell-cell fusion. Background fusion level was measured from effector BSR-T7 cells transfected with MojV-G only. Fold-change in RLU as compared with background fusion is shown. Data shown are mean values from five independent biological replicate experiments (*n*=5, error bars represent s.d.).

Key features for sialic acid binding and neuraminidase functionality in paramyxovirus HN glycoproteins include a conserved hexapeptide NRKSCS motif, triarginyl cluster (residues R174, R416 andR498 in NDV), and a tyrosine involved in neuraminidase activity and fusion promotion (Y526 in NDV)^26^. While sialic acid can be accommodated in the top face of the MojV-G β-propeller, in a cleft somewhat reminiscent to the sialic acid-binding pockets of avula-, respiro- and rubula-virus HN glycoproteins there is very limited sequence conservation between MojV-G and HN glycoproteins at these regions (Fig. 8a). For example, only two of the residues comprising the classical HN glycoprotein hexapeptide motif are conserved in MojV-G and none of the triarginyl cluster residues implicated in sialic acid binding is present (Fig. 8a). To assess the sialic acid dependence of MojV-F/G-mediated fusion, we tested the effect of kifunensine, an α-mannosidase inhibitor that inhibits the formation of sialylated complex-type *N*-linked glycans^33^, in a heterologous fusion assay. Although we cannot discount the presence of sialic acid presented by *O*-linked glycosylation, kifunensine treatment had a pronounced effect on the cell surface binding of a sialic acid specific lectin (PHA-L) (Supplementary Fig. 6), whereas fusion mediated by MojV-F/G was unaffected (Fig. 8b). We suggest that the absence of conserved residues involved in sialic acid recognition support a sialic-acid independent host cell entry mechanism for MojV.

## Discussion

The spillover of novel pathogens from animals into human populations is unpredictable and constitutes an ever-persistent burden to global health^50^. As realized from the recent Zika virus epidemic in Brazil^51^, emerging viruses, which historically are thought to exhibit little or no pathogenicity, can rapidly become established prominent pathogens. In this retrospective context, it is evident that examination of the pathobiology and tropism characteristics of emerging viruses such as MojV, before such outbreaks, will not only deepen our understanding about the determinants of cross-species infection but also strengthen our preparedness for potentially large-scale outbreaks in the future.

The impact of MojV infection in humans remains a mystery. Rodent-borne MojV was discovered in a cave following the deaths of miners in Yunnan Province, Southern China. Although it is unknown whether MojV was directly responsible for these fatalities, sequencing of the MojV genome establishes a relationship with highly pathogenic HNVs^1^, triggering significant cause for concern. Our investigation revealed that MojV-G is antigenically distinct from the currently recognized members of the HNV genus and does not interact with antibodies that target established HNV members. These data indicate that large-scale serological studies focused on detecting HNVs in human and animal reservoirs, such as that performed by Pernet *et al*.^7^ using HNV pseudoparticles, would probably fail to detect this or antigenically distal henipa viruses, posing a challenge in assessing the prevalence and impact of these viruses upon human health.

Given that HNV-G is a key determinant of host tropism^25^,^32^, we sought to establish whether MojV-G could be similarly utilized for viral transmission into human host cells. This analysis revealed that while human cell lines were identified as susceptible to MojV entry, MojV-G lacks the pathobiological features characteristic to the HNV genus, being incapable of interacting with ephrinB2 or ephrinB3 cell surface receptors (Fig. 3) or readily heterotypically complementing respective HNV-F glycoprotein partners (Fig. 6). Structural analysis provides a molecular rationale for this differential function. We observe that MojV-G lacks an ephrin-recognition site (Fig. 4), which is conserved among even the most genetically distant HNVs^30^. These data reveal that if MojV were to spillover into other organisms, such as humans, it would have to occur through an ephrinB2/B3-independent mechanism.

We also show that MojV is distinct from the other genera of the *Paramyxoviridae*. MojV-G is the only paramyxoviral glycoprotein to fall outside established receptor-specific structural groupings^16^, being nearly equidistant from the receptor-binding proteins of HNVs, morbilliviruses, and HN-displaying paramyxoviruses (Fig. 5). Furthermore, we show that MojV-G does not require *N*-linked glycosylation in the β-propeller domain (Fig. 4a and Supplementary Fig. 4). Thus, our structure not only shows that a paramyxoviral head domain can fold properly without *N*-linked glycans, but also broadens the known architectures of six**-**bladed β-propellers that can support paramyxovirus attachment and fusion triggering function. This structural individuality is suggestive that MojV lacks the functional constraints imposed upon characterized G-, H- and HN-glycoproteins and utilizes a novel, yet-unknown host receptor. Indeed, our functional data corroborate the independence of MojV host cell entry from known proteinaceous (ephrinB2/B3, Fig. 3 and CD150, Fig. 7) and carbohydrate paramyxovirus receptors (sialic acid, Fig. 8).

The structural, functional, and antigenic distinctiveness of MojV-G reveals MojV as something of an enigma and establishes a surprising pathobiological diversity among the members of the *Paramyxoviridae* family. Although MojV has been genetically classified as a HNV species^1^,^6^, the absence of conserved antigenic, architectural, and receptor-mediated host cell entry characteristics raises interesting questions about the evolutionary lineage and ancestral division of MojV from established African and Asiatic HNVs (for example, KV and NiV, respectively). Unlike morbilliviruses, which are unified in their common usage of proteinous CD150 as an entry receptor, the inability of MojV to recognize ephrinB2/B3 reveals significant gaps in our knowledge with regards to the prevalence and diversity of HNVs more generally. *In toto*, our data support the independent functional categorization of MojV from known protein and glycan receptor-specific paramyxoviruses and indicate an idiosyncratic mechanism of host-cell entry.

## Methods

### Protein expression and purification

A region including the MojV-G six-bladed β-propeller domain (residues 166–625, NCBI Reference Sequence YP_009094095) was PCR-amplified from codon-optimized cDNA (GeneArt, Life Technologies) and cloned into the mammalian expression vector pHLsec^52^. The resulting recombinant plasmids were used for transient expression in HEK 293T cells (ATCC CRL-1573) in the presence of the class 1 α-mannosidase inhibitor, kifunensine, at 5 μM^34^. Transient expression was performed using 2 mg DNA with 4 mg of the transfection reagent, polyethylenimine (Sigma-Aldrich 408727), per litre of cell culture. Transfected cultures were incubated for 90 h to allow expression, followed by supernatant diafiltration (QuixStand Benchtop System, GE Healthcare) before protein purification.

The recombinantly produced, soluble MojV-G was purified by immobilized metal-affinity chromatography (5 ml HisTrap FF crude column and ÄKTA FPLC system, GE Healthcare) at room temperature, using 250 mM imidazole for elution followed by size-exclusion chromatography using a Superdex 200 10/300 GL column (GE Healthcare), in 10 mM TRIS pH 8.0, 150 mM NaCl buffer. Purified protein was treated with EndoF_1_ (0.01 mg EndoF_1_ per 1 mg MojV-G, incubated for 18 h at 21 °C) to cleave glycosidic bonds of *N*-linked sugars within the di-*N*-acetylchitebiose core. Following deglycosylation, MojV-G purified by size-exclusion chromatography using a Superdex 200 10/300 GL column (GE Healthcare), in 10 mM TRIS pH 8.0, 150 mM NaCl buffer.

### Crystallization and structure determination

Crystals were grown using a sitting drop vapour diffusion method^53^ using 100 nl protein (5 mg ml^−1^) plus 100 nl precipitant at room temperature and appeared after 4–7 days in solution consisting of 16.4% (vol/vol) PEG 6000, 0.82 M lithium chloride and 0.082 M citrate pH 5.0. Crystals were flash frozen by immersion into a cryo-protectant containing 25% (vol/vol) ethylene glycol followed by rapid transfer into liquid nitrogen. X-ray diffraction data were recorded at beamline I24 (λ=0.96863 Å), Diamond Light Source, UK.

Data were indexed, integrated and scaled with XIA2/XDS^54^,^55^. Processing statistics are presented in Table 1. The structure of MojV-G was solved by molecular replacement using programs MRage and Phase and build within PHENIX^56^ using NiV-G (PDB accession number 2VSM) as the search model. Structure refinement was performed using iterative refinement using REFMAC^57^,^58^ and PHENIX^56^. Coot^59^ was used for manual rebuilding and MolProbity^60^ was used to validate the model.

Structural alignments and structure-based phylogeny analysis were performed with the programme SHP^61^,^62^ and molecular graphics images were generated using PyMOL (The PyMOL Molecular Graphics System, Version 1.7.0.3, Schrödinger, LLC). Domain schematics were produced using DOG 2.0 software^63^.

### Anti-KV monoclonal antibody generation

To produce anti-KV-G 1E10, BALB/c mice were sequentially immunized. The BALB/c mice were first genetically immunized by electroporation with mixtures of codon-optimized NiV matrix (M) protein, NiV-F and NiV-G expression plasmids, followed by NiV-M, HeV-F and HeV-G expression plasmids, and finally NiV-M, KV-F and KV-G expression plasmids. Vesicular stomatitis virus-ΔG pseudotyped with KV-F and KV-G (KVpp) was given via tail vein injection as the final protein boost before sacrifice and hybridoma production. Hybridomas were screened for anti-KV-G reactivity by both ELISA (using KVpp as the coated antigen) and flow cytometry of KV-G transfected cells.

### Anti-HNV monoclonal antibody staining

HEK 293T cells (ATCC CRL-3216) were transfected (using Lipofectamine LXT, Invitrogen) with mammalian expression vectors encoding either MojV-G-HA, NiV-G-HA, HeV-G-HA, KV-G-HA or an empty vector control. At 48 h post transfection, cells were scraped, washed in 2% (vol/vol) fetal bovine serum (FBS)/PBS and incubated with neat supernatant from hybridoma cell lines expressing rabbit monoclonal antibodies 26, 45 (well-characterized, cross-reactive anti-NiV-G/HeV-G monoclonal antibodies)^64^,^65^, 213 (a well-characterized, specific anti-NiV-G monoclonal antibody)^66^,^67^ or mouse monoclonal antibody 1E10 (an anti-KV-G monoclonal antibody, described above) for 1 h.

Following the primary stain, cells were washed in 2% (vol/vol) FBS/PBS and incubated with a phycoerythrin (PE)-conjugated goat anti-rabbit polyclonal antibody (pAb) (Invitrogen, catalogue number: P-2771MP) diluted 1:1,000 or allophycocyanin-conjugated goat-anti-mouse pAb (Jackson ImmunoResearch, catalogue number: 115-136-071) diluted 1:1,000. Cells were washed in 2% (vol/vol) FBS/PBS, PBS and then fixed in 2% (vol/vol) PFA/PBS and subjected to flow cytometry (BD Cantoll). Cell surface expression of each G protein was assessed using a primary rabbit anti-HA pAb (Novus, catalogue number: NB600-363) at 1:1,000 dilution and a secondary PE-conjugated goat-anti-rabbit pAb (Invitrogen, catalogue number: P-2771MP) at 1:1,000 dilution. All incubations were performed on ice and all washes were carried out at 4 °C.

### Soluble ephrinB2/B3-hFc staining

HEK 293T (ATCC CRL-3216) and CHO pgsA-745 (ATCC CRL-2242) cells were transfected (using Lipofectamine LTX) with mammalian expression vectors encoding either MojV-G-HA, NiV-G-HA or an empty vector. At 48 h post transfection, cells were scraped, washed in 2% (vol/vol) FBS/PBS and incubated with either human ephrinB2-hFc, ephrinB3-hFc or hFc (R&D Systems) at 10 nM in 2% (vol/vol) FBS/PBS for 1 h. Cells were washed, stained with a goat F(ab’)2 anti-human IgG-PE (Invitrogen, catalogue number: H10104) at a 1:1,000 dilution for 1 h in 2% (vol/vol) FBS/PBS, fixed and subjected to flow cytometry. EphrinB2 and B3 binding was adjusted for cell surface expression of each G protein by staining for a C-terminal HA epitope tag.

### Soluble CD150 staining

HEK 293T cells were transfected with plasmids expressing MojV-G-HA or MV-H glycoprotein (as a positive control), scraped 48 h post transfection, washed in 2% (vol/vol) FBS/PBS and incubated with soluble CD150 engineered with a C-terminal FLAG epitope tag generously provided by Phillipe Plattet^68^. Cells were washed, incubated with a mouse monoclonal anti-FLAG antibody diluted 1:2,000 (Sigma, catalogue number: F3165-1MG), washed and then stained with an Alexa 647-labelled goat anti-mouse antibody diluted 1:10,000 (Invitrogen, catalogue number: A-21236). Cells were fixed and subjected to flow cytometry.

### Syncytia and heterologous fusion assays

BSR-T7 (BHK-based cell line with stable expression of T7 polymerase)^69^ cells were transfected with plasmids encoding the MojV F and G glycoproteins in isolation or in combination with a variety of HNV glycoproteins or empty vector. Cells were monitored for syncytia formation by phase-contrast microscopy.

BSR-T7 cells (effector cells) were transfected (using Lipofectamine LTX) with expression plasmids encoding either the paired F and G glycoproteins, the F gene with pCAGGS or the G gene with pCAGGS across a panel of different HNVs. A549 (ATCC CCL-185), BHK-21 (ATCC CCL-10), U87 (ATCC HTB-14) and 293T (ATCC CRL-3216) target cells were transfected (using Lipofectamine LTX) with a plasmid encoding T7-driven *Gaussia* luciferase. The target cells were trypsinized 12 h post transfection. The various target cells were allowed to recover in media in a falcon tube for 12 h, resuspended in media and equal amounts were applied to each well of the BSR-T7-transfected effector cells. Supernatant was harvested at 48, 72, 96 and 120 h post-application of target cells and assayed for *Gaussia* luciferase (NEB *Gaussia* luciferase assay system). This assay was modified to include (1) target cells, including CHO pgsA-745 (ATCC CRL-2242) cells stably expressing ephrinB2; (2) soluble NiV-G-hFc or hFc incubated with target cells for 1 h on ice at a concentration of 20 μg ml^−1^ before application of the cells to effector cells; (3) treatment of target cells with 20 μM kifunensine for 24 h before their application to effector cells; and (4) BSR-T7 effector cells transfected with heterotypic (in addition to homotypic) combinations of plasmids encoding F and G glycoprotein from a panel of HNV and HNV-like viruses.

### Data availability

Coordinates and structure factors of MojV-G have been deposited in the Protein Data Bank with the accession code 5NOP. The data that support the findings of this study are available from the corresponding authors upon request.

## Additional information

**How to cite this article:** Rissanen, I. *et al*. Idiosyncratic Mòjiāng virus attachment glycoprotein directs a host-cell entry pathway distinct from genetically related henipaviruses. *Nat. Commun.* **8,** 16060 doi: 10.1038/ncomms16060 (2017).

**Publisher’s note**: Springer Nature remains neutral with regard to jurisdictional claims in published maps and institutional affiliations.

## Supplementary Material

Supplementary Information

## Figures and Tables

**Table 1:** Crystallographic data collection and refinement statistics for MojV-G.

	**MojV-G**
Data collection*	
Space group	*P*2_1_2_1_2_1_
Cell dimensions	
*a*, *b*, *c* (Å)	60.0, 102.3, 162.1
*α*, *β*, *γ* (°)	90.0, 90.0, 90.0
Resolution (Å)	49.3–1.94 (1.99–1.94)†
*R*_merge_	0.156 (0.820)
*I*/*σI*	8.7 (1.9)
Completeness (%)	99.7 (98.4)
Redundancy	5.2 (5.0)
	
*Refinement*	
Resolution (Å)	49.3–1.94 (1.97–1.94)
No. reflections	74,170 (3,627)
*R*_work_/*R*_free_	0.182 / 0.222
No. atoms	
Protein	6,683
Ligand/ion	6
Water	940
*B*-factors	
Protein	19.9
Ligand/ion	35.7
Water	27.8
R.m.s deviations	
Bond lengths(Å)	0.006
Bond angles (°)	0.782
Ramachandran statistics	
Residues in preferred region (%)	96.3
Residues in allowed region (%)	3.7
Outliers (%)	0

^*^Data were collected from two crystals and merged.

^†^Highest resolution shell is shown in parentheses.
