# Supplementary material for: Idiosyncratic Mòjiāng virus attachment glycoprotein directs a host-cell entry pathway distinct from genetically related henipaviruses
Source: Nat Commun. 2017 Jul 12;8:16060. doi: 10.1038/ncomms16060 (PMC5510225; doi:10.1038/ncomms16060)
Supplement: Supplementary Information [file ncomms16060-s1.pdf]

File Name: Supplementary Information
Description: Supplementary Figures, Supplementary Methods and Supplementary References

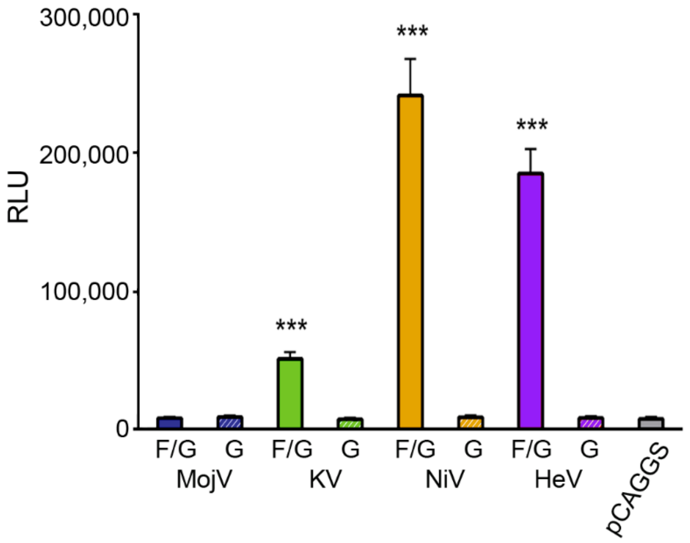

Supplementary Figure 1. EphrinB2 does not support Mòjiāng virus (MojV) F/G mediated fusion. BSR-T7 cells (effector cells) were transfected with expression plasmids encoding either paired fusion protein (F) and attachment glycoprotein (G), or the G protein with pCAGGS. The henipaviral glycoproteins used were from MojV (blue), Nipah virus (NiV, orange), Hendra virus (HeV, purple) and Kumasi virus (KV, green). CHO pgsA-745 target cells expressing ephrinB2 were transfected with a plasmid encoding T7-driven *Gaussia* luciferase. *Gaussia* luciferase activity, read in RLU (Relative Light Units), was used as a marker for cell-cell fusion. While MojV F/G expression does not result in fusion with ephrinB2-displaying target cells, henipaviral envelope proteins induce fusion efficiently. Statistical significance from the pCAGGS control was evaluated by a one-way ANOVA (*** $P < 0.001$). Data shown are mean values from five independent biological replicate experiments (n=5, error bars represent standard error, s.d.).

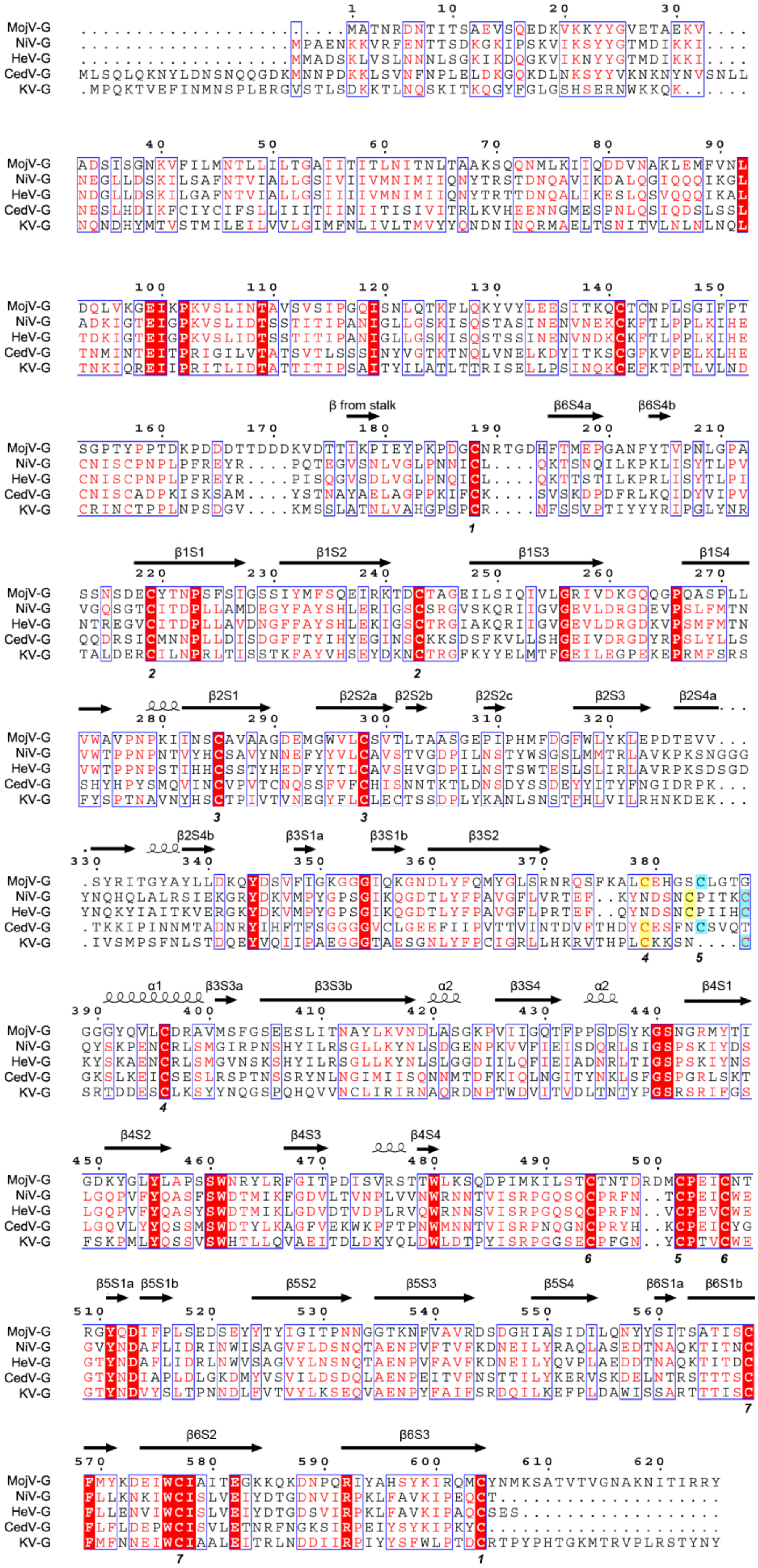
MojV-G
NiV-G
HeV-G
CedV-G
KV-G
β from stalk
β6S4a
β6S4b
β1S1
β1S2
β1S3
β1S4
β2S1
β2S2a
β2S2b
β2S2c
β2S3
β2S4a
β2S4b
β3S1a
β3S1b
β3S2
α1
β3S3a
β3S3b
α2
β3S4
β4S1
β4S2
β4S3
β4S4
β5S1a
β5S1b
β5S2
β5S3
β5S4
β6S1a
β6S1b
β6S2
β6S3

Supplementary Figure 2. Alignment of MojV glycoprotein G and henipaviral G protein sequences. Sequence alignment of MojV-G and attachment glycoprotein G from NiV, HeV, CedV, and KV. The alignment was prepared with MultAlin[1] and plotted with ESPRIPT[2]. Residues which are highlighted in red are fully conserved, residues which are colored red are partially conserved, and residues which are white are not conserved. Secondary structure elements of MojV-G are annotated with an arrow (β-strand) and helix (α-helix) and are labelled according to standard paramyxovirus nomenclature. Disulphide bonds 1 to 7 are indicated underneath the alignment, and while the sequence alignment does not align the N-terminal partners for disulphide bonds 4 and 5 across MojV-G and henipaviruses, disulphide bonds occupy conserved position structurally. These homologous residues in MojV- and henipaviral G proteins have been highlighted in yellow and light blue for disulphide bonds 4 and 5, respectively. NCBI Reference Sequence accession codes: MojV, YP_009094095.1; NiV, NP_112027.1; HeV, NP_047112.2; CedV, YP_009094086.1 and KV (GH-M74a), YP_009091838.1.

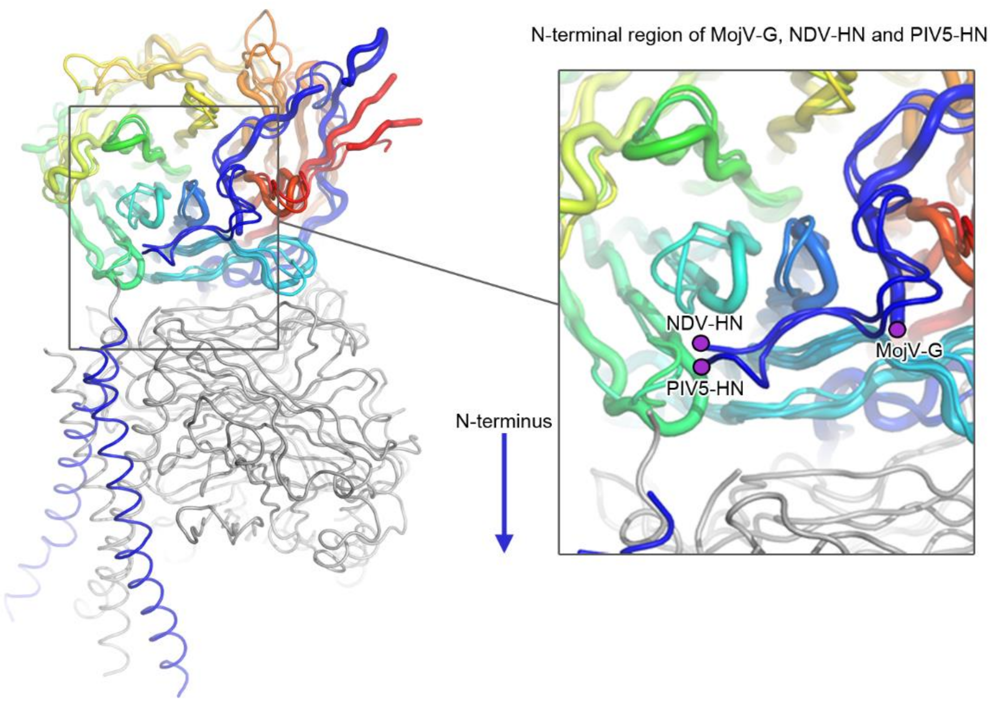

Supplementary Figure 3. MojV-G N-terminal region. The MojV-G N-terminal region aligns well with the N-terminal regions from full-length ectodomains from parainfluenza virus 5 HN (PIV5-HN; PDB accession code 4JF7) and Newcastle disease virus HN (NDV-HN; PDB accession code 3T1E) glycoproteins, indicating that full-length MojV-G might also exhibit similar tertiary arrangement. MojV-G, PIV5-HN, and NDV-HN are shown in tube representation colored blue-to-red rainbow from the N-terminus to the C-terminus with MojV-G rendered thicker than the others. Dimeric subunit protomers in heads-down orientation from NDV-HN and PIV5-HN are shown in gray. The crystallized construct of MojV-G included residues 166-625, of which residues 175-615 were ordered in the crystal structure.

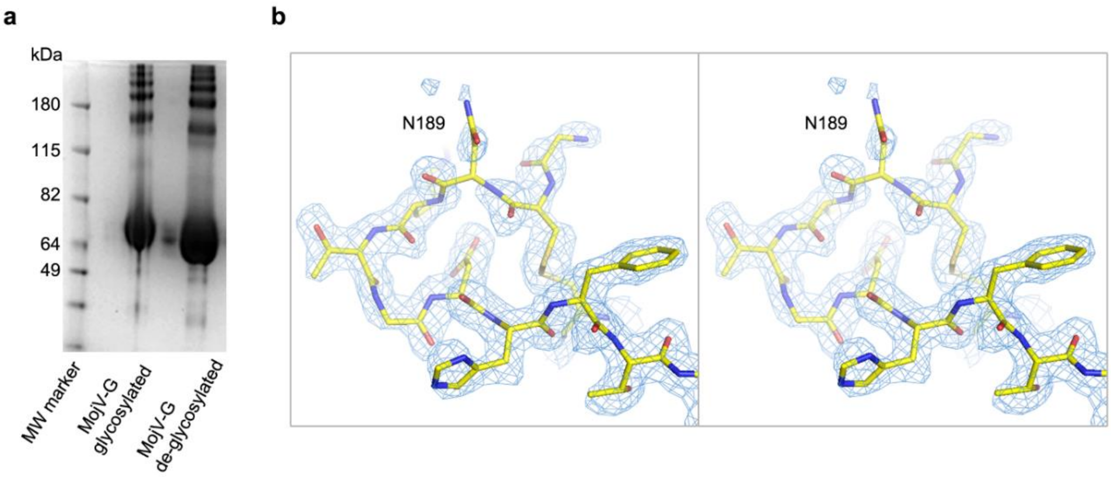

Supplementary Figure 4. *N*-linked glycosylation in the crystallized MojV-G β-propeller domain. (**a**) SDS-PAGE analysis of MojV-G prior to and after deglycosylation with endoglycosidase F_1 (left and right bands, respectively) shows a band shift associated with deglycosylation. Deglycosylated protein was pooled and set up for crystallization following size-exclusion chromatography. (**b**) Stereo-view of the region around MojV-G *N*-linked glycosylation site N189. Molecule backbone representation of MojV-G residue N189, the only *N*-linked glycosylation sequon ordered in the crystal, with a *2Fo-Fc* electron density map contoured at 1σ, shows the absence of GlcNAc-derived density. This region of the peptide is flexible and evidence for the residue N189 is only observed in one of the two molecules observed in the asymmetric unit (chain B).

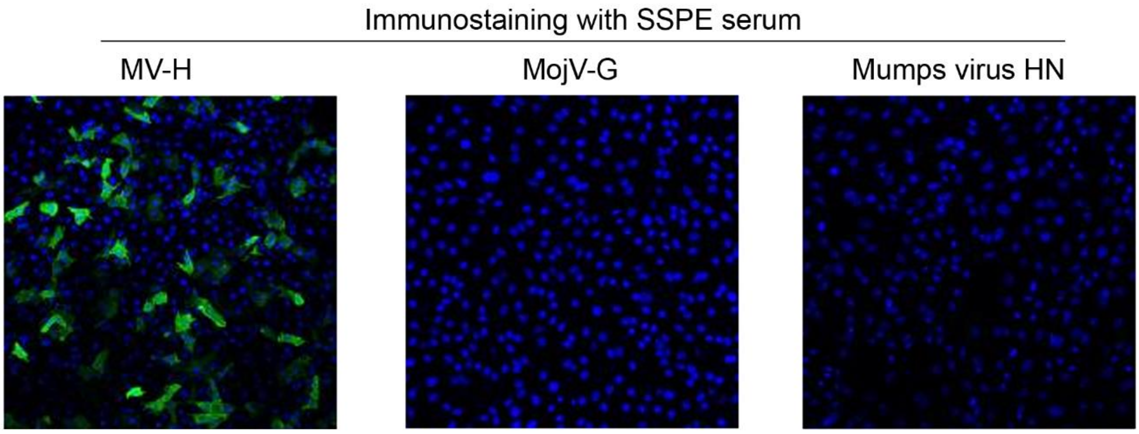

Supplementary Figure 5. Antibodies against measles virus (MV) do not recognize MojV-G. Vero cells transfected with MV (KS strain) hemagglutinin protein (H), MojV-G, or Mumps virus (G09 strain) hemagglutinin-neuraminidase (HN) expressing plasmid were immunostained with human subacute sclerosing panencephalitis (SSPE) serum. Binding of SSPE antibodies was detected with goat anti-human IgG Alexa Fluor 488 conjugate, nuclei were counterstained with 4',6-diamidino-2-phenylindole and cells were visualized by confocal scanning laser microscopy. Results show that only cells displaying MV-H are detected by anti-MV antibodies in SSPE serum, establishing MojV-G as antigenically distinct from MV. Mumps HN is included as a negative control.

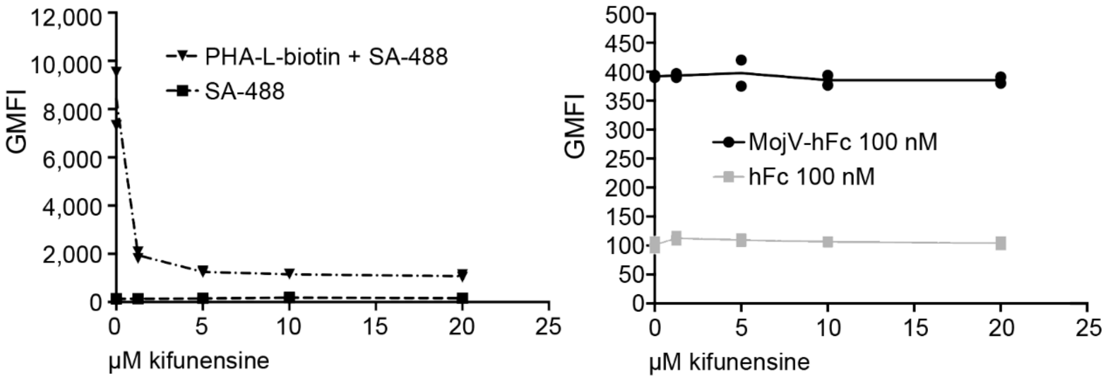

Supplementary Figure 6. Kifunensine does not affect the binding of MojV-G to target cells. Treatment of Vero target cells with kifunensine, an α-mannosidase inhibitor that prevents the formation of sialylated complex-type *N*-linked glycans, effectively reduced the binding of sialic acid specific lectin, PHA-L, at kifunensine concentrations as low as 1.25 μM (left panel), while the binding of soluble MojV-G-hFc was unaffected by kifunensine treatment (right). Data shown are the individual geometric mean fluorescence intensity (GMFI) values from two independent biological replicate experiments (n=2). Lines connect the means of each set of duplicates. Each individual GMFI value was generated from a composite of 10,000 collected events.

Supplementary Methods

SSPE serum immunostaining. Vero cells (ATCC CRL-1586) were transfected for 48 hours with plasmids expressing MojV-G, MV-KS strain H[3] or mumps virus-G09 strain HN glycoproteins. Antigens were visualized by indirect immunofluorescence using human subacute sclerosing panencephalitis (SSPE) serum and a primary antiserum and goat anti-human IgG (H+L) Alexa Fluor® 488 conjugate (Invitrogen, Catalog#: A-11013) as a secondary antibody. Nuclei were counterstained with 4',6-diamidino-2-phenylindole (DAPI) and cells were visualized by confocal scanning laser microscopy, as previously described[4]. Results from this experiment are shown in **Supplementary Figure 5**.

Kifunensine treatment of cells. Vero (ATCC CRL-1586) and BSR T7 (BHK-based cell line with stable expression of T7 polymerase)[5], cells were treated with kifunensine (from 0 to 20 µM) for 24 hours. Cells were scraped, washed in 2% (vol/vol) FBS/PBS and incubated with either 100 nM of MojV-G-hFc, hFc, or L-PHA in 2% (vol/vol) FBS/PBS for one hour. Cells were washed in 2% (vol/vol) FBS/PBS and incubated with either goat anti-hFc conjugated with PE (Invitrogen, Catalog#: H10104) or SA-488 at 1:1000 for one hour in 2% (vol/vol) FBS/PBS. Cells were washed, fixed, and subjected to flow cytometry. Results from this experiment are shown in **Supplementary Figure 6**.

Supplementary References

1. Corpet, F. Multiple sequence alignment with hierarchical clustering. *Nucleic Acids Res* **16**, 10881-90 (1988).
2. Gouet, P., Courcelle, E., Stuart, D.I. & Metoz, F. ESPript: analysis of multiple sequence alignments in PostScript. *Bioinformatics* **15**, 305-308 (1999).
3. Lemon, K. et al. Early target cells of measles virus after aerosol infection of non-human primates. *PLoS Pathog* **7**, e1001263 (2011).
4. Duprex, W.P., McQuaid, S., Hangartner, L., Billeter, M.A. & Rima, B.K. Observation of measles virus cell-to-cell spread in astrocytoma cells by using a green fluorescent protein-expressing recombinant virus. *J Virol* **73**, 9568-9575 (1999).
5. Buchholz, U.J., Finke, S. & Conzelmann, K.K. Generation of bovine respiratory syncytial virus (BRSV) from cDNA: BRSV NS2 is not essential for virus replication in tissue culture, and the human RSV leader region acts as a functional BRSV genome promoter. *J Virol* **73**, 251-9 (1999).
